# An unusual case of an ulcerative colitis flare resulting in disseminated intravascular coagulopathy and a bladder hematoma: a case report

**DOI:** 10.1186/1471-230X-4-26

**Published:** 2004-10-07

**Authors:** David L Suskind, Karen Murray, Dennis Christie

**Affiliations:** 1 Department of Pediatrics, Seattle Children's Hospital and Regional Medical Center, University of Washington, 4800 Sand Point Way NE, Seattle, Washington, USA

## Abstract

**Background:**

Disorders of coagulation have long been associated with inflammatory bowel disease. Children, as well as adults, with both active and inactive ulcerative colitis have been found to have abnormal coagulation and fibrinolysis. Disseminated intravascular coagulation arises from an overwhelming of the haemostatic regulatory mechanisms leading to an excessive generation of thrombin and a failure of the normal inhibitory pathways to prevent systemic effects of this enzyme. Ulcerative colitis has been associated with disseminated intravascular coagulation in conjunction with septicemia, toxic megacolon and surgery.

**Case presentation:**

A fourteen-year-old boy with a history of poorly controlled ulcerative colitis presented with nonbilious emesis, hematochezia, and hematuria. Laboratory workup revealed disseminated intravascular coagulation. He was placed on triple antibiotics therapy. An infectious workup came back negative. A computerized tomography (CT) scan of the abdomen revealed a marked thickening and irregularity of the bladder wall as well as wall thickening of the rectosigmoid, ascending, transverse, and descending colon. Patient's clinical status remained stable despite a worsening of laboratory values associated with disseminated intravascular coagulation. Patient was begun on high dose intravenous steroids with improvement of the disseminated intravascular coagulation laboratory values within 12 hours and resolution of disseminated intravascular coagulopathy within 4 days. A thorough infectious workup revealed no other causes to his disseminated intravascular coagulation.

**Conclusions:**

The spectrum of hypercoagulable states associated with ulcerative colitis varies from mild to severe. Although disseminated intravascular coagulation associated with ulcerative colitis is usually related to septicemia, toxic megacolon or surgery, we present a case of an ulcerative colitis flare resulting in disseminated intravascular coagulation and a bladder hematoma.

## Background

A wide variety of disorders are associated with the development of disseminated intravascular coagulation (DIC). Initiation usually involves mechanical tissue injury and or endothelial cell activation and injury. DIC arises from an overwhelming of the haemostatic regulatory mechanisms leading to an excessive generation of thrombin and a failure of the normal inhibitory pathways to prevent systemic effects of the enzyme leading to DIC [1]. Ulcerative colitis has been associated with DIC. In previously reported cases, DIC has arisen from active disease in conjunction with septicemia, toxic megacolon or surgery [2-5]. The authors report a pediatric case of DIC associated with a colitis flare resulting in a bladder hematoma.

## Case presentation

A 14-year-old boy with a diagnosis of ulcerative colitis based on colonic histology, serology and a normal barium study of his small bowels was admitted with a five-day history of nonbilious vomiting and bloody diarrhea. Additional symptoms included recent onset hematuria, and low-grade fevers to 100.4 C over the prior four days. He had also sustained a 25 lb weight loss in the last six months, indicating a lack of disease control.

As an outpatient, his maintenance therapy included mesalamine (1 gram three times a day), and mercaptopurine (75 mg once per day). In addition, he had been started on prednisone approximately 7 weeks prior for treatment of an ulcerative colitis flare. His current dose of prednisone was 10 mg once a day. Soon after symptoms begun, he had been placed on ciprofloxacin as treatment for a presumptive flare.

Physical exam showed he was afebrile, with a heart rate of 130 beats per minute, respiratory 16 breaths per minute and blood pressure 115/67 mmHg. He was alert although with a sallow appearance. Abdominal exam revealed a soft nontender nondistended abdomen. Rectal showed normal external exam with grossly bloody stool. Initial blood work showed hemoglobin of 12.3, a normal white blood cell count, normal differential and normal platelet count with a mildly elevated prothrombin time of 16.2 with an international normalized ratio (INR) of 1.2. Urine analysis showed a specific gravity of 1.035, 3+blood, +ketones and > 100 RBC per high powered field and 0–5 WBC per high power field. Abdominal ultrasound revealed irregular shaped bladder wall.

Patient was placed on intravenous fluids (IV) as well as metronidazole (IV). Blood and urine cultures were sent for analysis. Stool was sent for culture and for *Clostridium difficile *toxin analysis.

**Figure 1:**
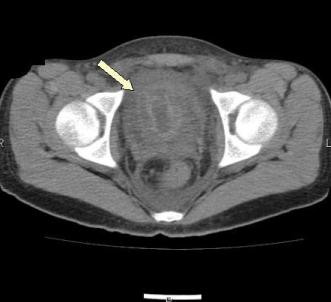
Abdominal CT revealing a marked thickening and irregularity of the bladder wall consistent with bladder hematoma.

Serial repeat lab works the following day revealed a dropping hemoglobin (7.4 g/dL) and platelet count (64 K/mm^3^) increasing PT/PTT (21.3/47 seconds) with an INR of 1.8. Blood smear showed moderate amount of elliptocytes, schistocytes, microcytes and fragmented red blood cells. Initial DIC panel revealed an elevated D-dimer of 4.9 mcg/mL with a normal thrombin time and fibrinogen. Thrombin time subsequently increased to > 120 seconds. D-dimers increased to 10.3 mcg/mL. A computerized tomography (CT) scan of the abdomen revealed a marked thickening and irregularity of the bladder wall as well as wall thickening of the rectosigmoid, ascending, transverse, and descending colon (Figure 1). Urology was consulted and felt that this represented a submucosal hematoma.

**Figure 2:**
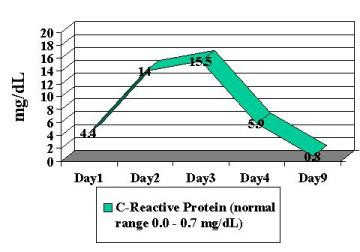
Graphic illustration of C-reactive protein throughout hospitalization: Day 1 (admission date) – Day 9 (day of discharge). Patient received intravenous steroids at approximately 36 hours into hospitalization.

**Figure 3:**
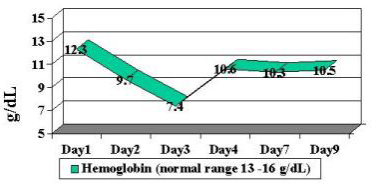
Graphic illustration of hemoglobin throughout hospitalization: Day 1 (admission date) – Day 9 (day of discharge). Patient received intravenous steroids at approximately 36 hours into hospitalization.

**Figure 4:**
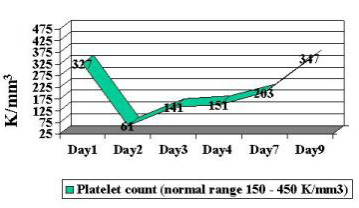
Graphic illustration of platelets throughout hospitalization: Day 1 (admission date) – Day 9 (day of discharge). Patient received intravenous steroids at approximately 36 hours into hospitalization.

**Figure 5:**
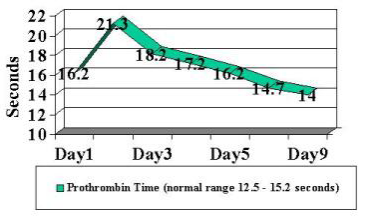
Graphic illustration of prothrombin time throughout hospitalization: Day 1 (admission date) – Day 9 (day of discharge). Patient received intravenous steroids at approximately 36 hours into hospitalization.

**Figure 6:**
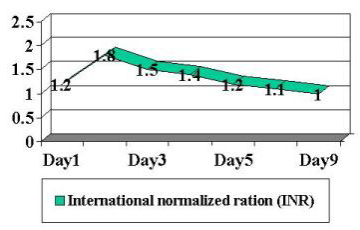
Graphic illustration of international normalized ratio throughout hospitalization: Day 1 (admission date) – Day 9 (day of discharge). Patient received intravenous steroids at approximately 36 hours into hospitalization.

**Figure 7:**
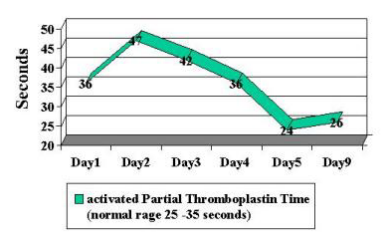
Graphic illustration of partial thromboplastin time C-reactive protein throughout hospitalization: Day 1 (admission date) – Day 9 (day of discharge). Patient received intravenous steroids at approximately 36 hours into hospitalization.

Patient was begun on broad-spectrum antibiotics because of concerns regarding possible bacteremia and a worsening DIC laboratory picture. Blood, stool and urine cultures returned negative. Viral cultures and monoclonal antibody staining for adenovirus detection in the urine was negative. Despite a worsening in the DIC panel, the patient remained clinically unchanged. IV steroids were begun approximately 36 hours into patient's hospital stay. Patient had a stabilization of PT/PTT/INR/thrombin time and D-dimer, and a subsequent normalization of labs over the following 4-day period ( Figure 2, 3, 4, 5, 6, 7, 8 ). Patient's diarrhea and hematuria resolved as well. Colonscopy revealed chronic colitis consistent with ulcerative colitis. Cystoscopy revealed a fibrin clot consistent with submucosal hematoma. Patient was discharged from the hospital on a steroid taper, and remains in remission to date.

## Conclusions

Disorders of coagulation have long been associated with inflammatory bowel disease [6-11]. Children, as well as adults, with both active and inactive ulcerative colitis have been found to have abnormal coagulation and fibrinolysis[11]. It is unclear whether this is a direct or indirect result of inflammatory bowel disease.

Although hypocoagulable states have been noted in the literature, most studies indicate an associated hypercoagulable state. There appears to be an increase in thrombin-anti-thrombin complex and a decrease in antithrombin III activity, which causes an increase in thrombin generation[10,12,13]. Other studies have demonstrated an increase in fibrinogen content, increase Factor VIII, and Factor IX activity, platelet count and aggregation rate[9,12]. These hypercoagulable abnormalities return towards normal with therapy in direct correlation with sedimentation rate and clinical disease activity [12], but can still show mild abnormalities despite clinical remission[14].

The hypercoagulable state in ulcerative colitis is associated thromboembolic events; although uncommon, deep vein thrombosis, pulmonary embolisms and stroke have been associated with ulcerative colitis[6,15-18]. Disseminated intravascular coagulopathy is a rare occurrence in inflammatory bowel disease. When it occurs, it is usually associated with other co-founding problems such as septicemia, toxic megacolon or surgery.

Presented is a case of DIC associated solely with an ulcerative colitis flare resulting in a bladder hematoma. We presume that the occurrence of DIC in this patient resulted from an acute flare on top of a chronic unremitting course of ulcerative colitis. A thorough infectious work-up of this patient did not reveal any infectious etiology that would have predisposed him to develop DIC. The presumed cause of the DIC was damage to the endothelial wall of the colonic blood vessels, which exposed blood to excessive amounts of tissue factor. This in turn led to the excessive generation of thrombin and a failure of the normal coagulation inhibitory pathways. By treating the ulcerative colitis flare, we decreased the intestinal inflammation and thereby decreased the endothelial cell damage. This, theoretically, resolved the DIC. Patient's clinical symptoms and laboratory values normalized after treatment with intravenous steroids, completely resolving the disseminated intravascular coagulopathy.

## Competing interests

The authors declare that they have no competing interests.

## Authors' contributions

DLS drafted the manuscript. KM and DC participated in the manuscript preparation. All authors approved the final manuscript.

**Figure 8:**
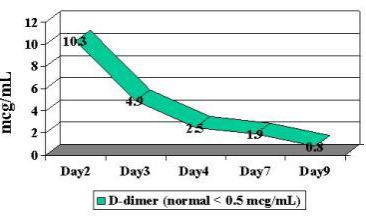
Graphic illustration of D-dimer throughout hospitalization: Day 1 (admission date) – Day 9 (day of discharge). Patient received intravenous steroids at approximately 36 hours into hospitalization.

## Pre-publication history

The pre-publication history for this paper can be accessed here:


